# Effect of sacubitril–valsartan on left ventricular remodeling in patients with acute myocardial infarction after primary percutaneous coronary intervention: a systematic review and meta-analysis

**DOI:** 10.3389/fphar.2024.1366035

**Published:** 2024-05-28

**Authors:** Yiheng Liu, Yue Sun, Weiran Dai

**Affiliations:** ^1^ Department of Cardiology, The Second Affiliated Hospital of Chongqing Medical University, Chongqing, China; ^2^ Department of Endocrinology, The First Affiliated Hospital of Chongqing Medical University, Chongqing, China

**Keywords:** sacubitril–valsartan, acute myocardial fraction, primary PCI, left ventricular remodeling, meta-analysis

## Abstract

**Background:**

Sacubitril–valsartan has been widely reported for reducing the risk of cardiovascular death and improving left ventricular remodeling in patients with heart failure (HF). However, the effect of sacubitril–valsartan in patients with acute myocardial infarction (AMI) remains controversial. Therefore, we conducted this meta-analysis to investigate whether sacubitril–valsartan could reverse left ventricular remodeling and reduce cardiovascular adverse events in AMI patients after primary percutaneous coronary intervention (PPCI).

**Materials and methods:**

Two researchers independently retrieved the relevant literature from PubMed, Embase, The Cochrane Library, China National Knowledge Infrastructure (CNKI), and the Wanfang database. The retrieval time was limited from inception to 1 June 2023. Randomized controlled trials (RCTs) meeting the inclusion criteria were included and analyzed.

**Results:**

In total, 21 RCTs involving 2442 AMI patients who underwent PPCI for revascularization were included in this meta-analysis. The meta-analysis showed that compared with the angiotensin-converting enzyme inhibitors (ACEI)/angiotensin receptor blockers (ARB), sacubitril–valsartan treatment in AMI patients after PPCI significantly reduced left ventricular end-diastolic dimension (LVEDD) (weighted mean difference (WMD) −3.11, 95%CI: −4.05∼−2.16, *p* < 0.001), left ventricular end-diastolic volume (LVEDV) (WMD −7.76, 95%CI: −12.24∼−3.27, *p* = 0.001), left ventricular end-systolic volume (LVESV) (WMD −6.80, 95%CI: −9.45∼−4.15, *p* < 0.001) and left ventricular end-systolic dimension (LVESD) (WMD −2.53, 95%CI: −5.30–0.24, *p* < 0.001). Subgroup analysis according to the dose of sacubitril–valsartan yielded a similar result. Meanwhile, PPCI patients using sacubitril–valsartan therapy showed lower risk of major adverse cardiac events (MACE) (OR = 0.36, 95%CI: 0.28–0.46, *p* < 0.001), myocardial reinfarction (OR = 0.54, 95%CI: 0.30–0.98, *p* = 0.041) and HF (OR = 0.35, 95%CI: 0.26–0.47, *p* < 0.001) without increasing the risk of renal insufficiency, hyperkalemia, or symptomatic hypotension. At the same time, the change of LV ejection fraction (LVEF) (WMD 3.91, 95%CI: 3.41–4.41, *p* < 0.001), 6 min walk test (6MWT) (WMD 43.56, 95%CI: 29.37–57.76, *p* < 0.001) and NT-proBNP level (WMD −130.27, 95%CI: −159.14∼−101.40, *p* < 0.001) were statistically significant.

**Conclusion:**

In conclusion, our meta-analysis indicates that compared with ACEI/ARB, sacubitril–valsartan may be superior to reverse left ventricular remodeling, improve cardiac function, and effectively reduce the risk of MACE, myocardial reinfarction, and HF in AMI patients after PPCI during follow-up without increasing the risk of adverse reactions including renal insufficiency, hyperkalemia, and symptomatic hypotension.

## Introduction

Acute myocardial infarction (AMI) is a common cardiovascular disease with high mortality caused by the rapid reduction of the coronary blood supply of the infarct-related artery (IRA). Primary percutaneous coronary intervention (PPCI) is an important coronary artery reperfusion treatment method for AMI that lowers the mortality of AMI (Ellen et al., 2003). Left ventricular remodeling reflects the heart’s maladaptation to mechanical, neurohormonal, and inherited changes by regulating ventricular size, shape, and function (Stefan et al., 2022). Timely PPCI can effectively guarantee the blood perfusion of the cardiomyocytes and rescue dying myocardium, but some AMI patients still develop left ventricular remodeling, including significant left ventricle (LV) enlargement and LV systolic dysfunction after coronary intervention (Kieran et al., 2020). According to statistics, up to 30% of patients with ST-elevated myocardial infarction (STEMI) develop left ventricular remodeling (Alessandro et al., 2020).

It is well established that pathological left ventricular remodeling post-myocardial infarction is correlated to the risk of heart failure (HF), which remains a major public health problem worldwide. In other words, effective inhibition of left ventricular remodeling may be a potential therapeutic direction to reduce the risk of HF after AMI in addition to timely revascularization. Recent evidence indicates that the deleterious effect of inappropriate activation of the neurohumoral system, including excessive activation of the sympathetic nervous system (SNS) and renin–angiotensin–aldosterone system (RAAS), contributes to adverse left ventricular remodeling and promotes HF development after AMI (Pfeffer et al., 1992; Liza et al., 2019). Despite the lack of drugs targeting left ventricular remodeling, neurohumoral antagonists such as angiotensin-converting enzyme inhibitors (ACEIs), angiotensin receptor blockers (ARBs), beta-blockers, and mineralocorticoid receptor antagonists (MRAs) serve as the cornerstone of present-day pharmacological HF treatment and also play an important role in attenuating myocardial remodeling (Marc et al., 2003; Robert et al., 2004; Theresa et al., 2021).

Sacubitril–valsartan is the first kind of angiotensin-receptor–neprilysin inhibitor (ARNI) that augments natriuretic peptides by inhibiting their breakdown by neprilysin, thereby counteracting SNS activation. Due to its dual inhibition mechanism, sacubitril–valsartan has a stronger effect on vasodilation, diuresis, and inhibiting myocardial hypertrophy than traditional ACEI/ARB (Duncan, 2016). In the PARADIGM-HF study, compared with enalapril, sacubitril–valsartan reduced the risk of HF hospitalization and cardiovascular death in HF patients with reduced ejection fraction (HFrEF) (John et al., 2014). Moreover, the latest ESC/AHA guidelines for HF endorse sacubitril–valsartan over traditional ACEI/ARB for managing chronic HFrEF, aiming to lower both morbidity and mortality (Heidenreich et al., 2022; McDonagh et al., 2024). As for HFpEF, sacubitril–valsartan has been demonstrated to alleviate left ventricular remodeling in rat models, with the results verified through cardiac color Doppler ultrasonography (Yu et al., 2023).

As mentioned above, left ventricular remodeling is associated with HF after AMI. Some clinical trials suggested that sacubitril–valsartan can further improve left ventricular remodeling and significantly reduce the risk of major adverse cardiac events (MACE) and HF rehospitalization in patients with AMI (Yi et al., 2020; Ahmed et al., 2021). However, the clinical benefits of using sacubitril–valsartan in patients with AMI remain controversial. An experience produced by Zhou and his colleagues showed that the application of sacubitril–valsartan did not decrease the incidence of cardiac death, myocardial infarction reoccurrence, and arrhythmia after acute myocardial infarction (Yang P. et al., 2022). Moreover, recent studies suggested that sacubitril–valsartan neither effectively improved left ventricular remodeling nor significantly reduced the risk of cardiovascular events in AMI patients (Kieran et al., 2021; Marc et al., 2021). In addition, few studies have directly compared the efficacy of sacubitril–valsartan to ACEI/ARB with respect to left ventricular remodeling and clinical benefits in those patients.

In summary, AMI is characterized by high morbidity and mortality. Timely PPCI for revascularization has improved survival rates of AMI patients, but some patients still develop left ventricular remodeling, which correlates to the risk of HF after PPCI. In order to better treat patients with AMI, especially to prevent and improve left ventricular remodeling after PPCI and the associated risk of HF, there is an urgent need for relevant potential drug therapy. Sacubitril–valsartan is widely used in the treatment of HF and has been found to reduce the risk of HF rehospitalization. Combined with the current clinical studies results, we hypothesized that sacubitril–valsartan treatment after PPCI in patients with AMI would help improve left ventricular remodeling and reduce the risk of cardiovascular events. However, its role in preventing the aforementioned changes is contentious based on existing research. Hence, we performed this meta-analysis of related randomized controlled trials (RCTs) to explore the effect of sacubitril–valsartan and ACEI/ARB treatment on left ventricular remodeling and reduction of cardiovascular adverse events in AMI patients after PPCI to provide guidance for clinical application.

## Materials and methods

### Literature search strategy

We designed this study according to the Preferred Reporting Items for Systematic Reviews and Meta-Analyses (PRISMA) indications for systematic reviews and meta-analyses (David et al., 2015). Two researchers (Yiheng Liu and Yue Sun) independently retrieved the relevant literature from PubMed, Embase, The Cochrane Library, China National Knowledge Infrastructure (CNKI), and the Wanfang database with the following MeSH terms: sacubitril-valsartan, LCZ696, Entresto, neprilysin inhibitor, ARNI, MI, AMI, STEMI, NSTEMI, PPCI, and emergency PCI. We restricted the retrieval time from inception to 1 June 2023. The literature language was restricted to English and Chinese. The reference lists of the retrieved studies were also checked to obtain potentially relevant studies. If the same study was reported by multiple journals, we included the most recent publication in our research for analysis.

### Literature selection

After the initial retrieval, we selected articles that met the inclusion criteria through intensive reading of the full text and included them in our research for data analysis. The inclusion criteria are as follows: 1) RCTs; 2) all patients were older than 18 years; 3) all patients met the AMI diagnostic criteria recommended by the newest American College of Cardiology/American Heart Association guidelines and underwent PPCI treatment to complete revascularization; 4) the experimental group was treated with sacubitril/valsartan on the basis of conventional treatment strategies, while the control group was treated with ACEI/ARB, and the rest of the treatments were the same as the experimental group; 5) articles reported the primary or secondary outcomes. Articles were excluded if they met the following exclusion criteria: 1) conference abstracts for which full-text information could not be obtained; 2) incomplete data or no access to original data literature; 3) the research objects were animals. Any disagreements are discussed and then submitted to the third researcher (Weiran Dai) for a final decision.

### Data extraction and quality assessment

Two researchers (Yiheng Liu and Yue Sun) independently extracted the data from the included articles and cross-checked the extracted data to ensure accuracy. Data extracted from the literature included the following: basic data of included literature (first author, year of publication, sample size, mean age, and male/female ratio), characteristics of patients (type of MI, concomitant HF or not), sacubitril–valsartan and ACEI/ARB treatments (drug name, initial time, dosage, frequency, and follow-up time), primary outcomes (echocardiographic indexes related to left ventricular remodeling including change of left ventricular end-diastolic dimension (LVEDD), left ventricular end-systolic dimension (LVESD), left ventricular end-diastolic volume (LVEDV) and left ventricular end-systolic volume (LVESV), incidence of MACE, myocardial reinfarction and HF during follow-up, and secondary outcomes (N-terminal pro-brain natriuretic peptide (NT-proBNP) level, change of LVEF, 6 min walk test (6MWT) distance, and incidence of adverse drug reactions including renal insufficiency, hyperkalemia, and symptomatic hypotension during follow-up). The risk of bias in the included articles was assessed by the Cochrane Collaboration bias risk assessment tool recommended by the Cochrane Handbook, which generally grades each domain of potential bias as “low risk,” “high risk,” or “unclear risk” (Zeng et al., 2015).

### Statistical analysis

In the current research, STATA 16.0 software was used for data analysis. RevMan 5.4 software was applied in the process of assessing the risk of bias. The categorical data are presented as the odds ratios (OR) and 95% confidence intervals (CI), while the continuous data are presented as weighted mean difference (WMD) and 95% CI. The extent of possible heterogeneity among included articles was assessed by the *I* ^*2*^ test. *I* ^*2*^ values in the 0%–25%, 25%–50%, 50%–75%, and 75%–100% ranges represent not important, mild heterogeneity, moderate heterogeneity, and considerable heterogeneity, respectively. The fixed effect model was used for statistical pooling when there was non-significant heterogeneity (*I* ^*2*^ < 50%); otherwise, a random effect model was used in the meta-analysis to reduce the bias of our research. Sensitivity analyses were conducted to explore the possible sources of heterogeneity. Additionally, we evaluated the publication bias by using the funnel plots and quantified the results by applying Egger’s regression test. Because we included studies consisting of patients of different characteristics, the participants were divided into two different groups according to the dose of the sacubitril–valsartan (sacubitril–valsartan maximum tolerated dose (MDT) or 200 mg bid V.S. sacubitril–valsartan 100 mg bid or less) for subgroup analyses. We set the *p*-value < 0.05 as the statistically significant level.

## Results

### Literature search results

**FIGURE 1:**
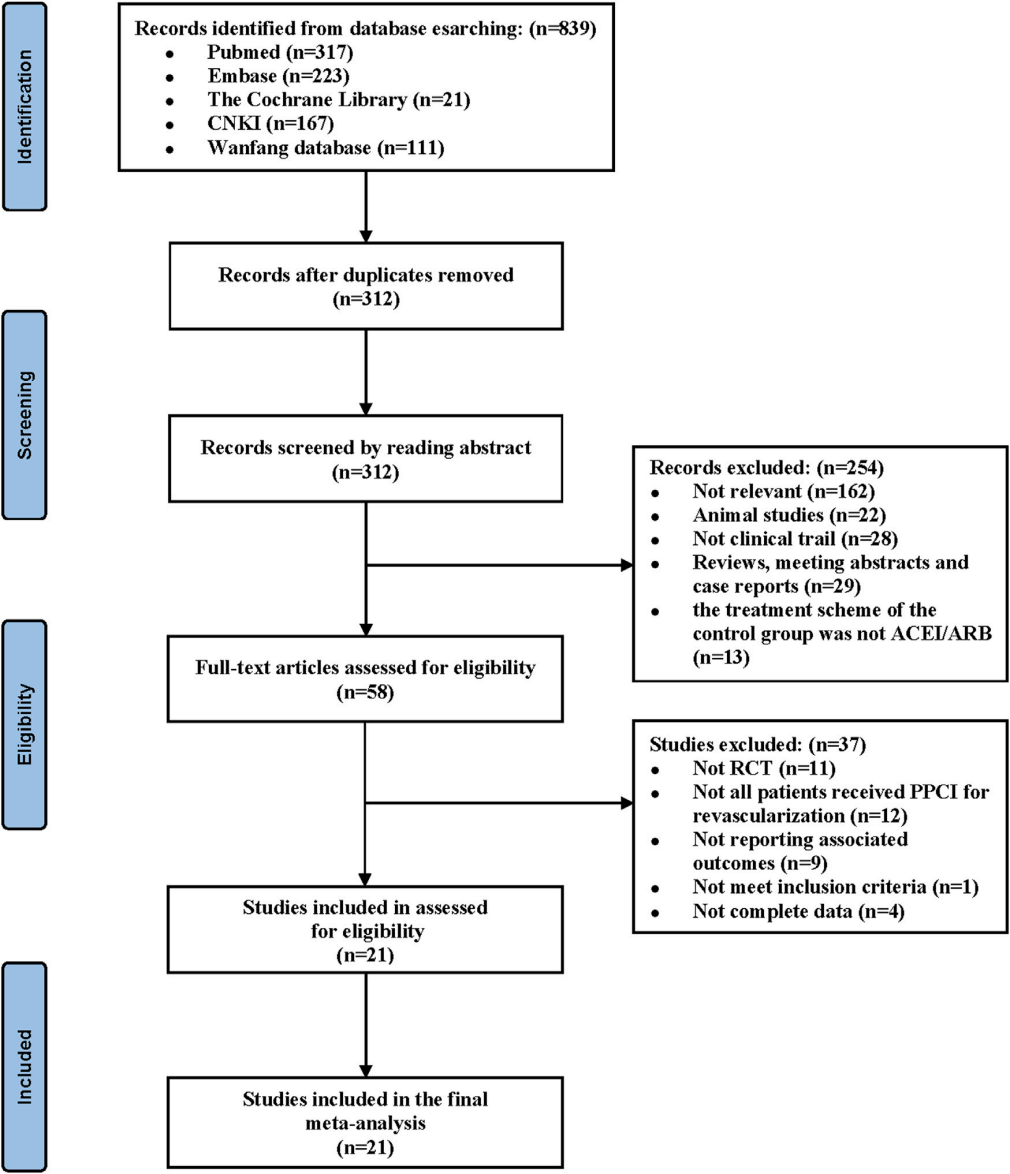
Flow diagram of the literature selection strategy. CNKI, China National Knowledge Infrastructure. RCT, randomized controlled trial. ACEI, angiotensin-converting enzyme inhibitor. ARB, angiotensin receptor blocker. PPCI, primary percutaneous coronary intervention.

According to the literature search strategy, we identified 839 relevant articles for eligibility by the title and abstract level. Ultimately, 21 RCTs with a total of 2,442 patients with AMI met the inclusion criteria and were included in the current meta-analysis (Chen et al., 2019; Chen et al., 2020; Dong et al., 2020; Li et al., 2020; Wang et al., 2020; Zhao, 2020; Zhao et al., 2020; Zhang et al., 2021a; Zhang et al., 2021b; Chen, 2021; Zhang R. et al., 2021; Gu, 2021; Rezq et al., 2021; Wang and Fu, 2021; Yang et al., 2021; Yang M. et al., 2022; Dong et al., 2022; Fu and Xu, 2022; Li and Xiong, 2022; Liu et al., 2022; Ma and Yang, 2022). The article screening process and results are shown in Figure 1. Among them, 1,209 patients received sacubitril–valsartan treatment after PPCI, while others received ACEI/ARB treatment. The baseline characteristics, such as sample size, mean age, and sex ratio of each study, were not significantly different between the two groups in each article. The mean follow-up duration ranged from 1 to 6 months. The basic characteristics of the included articles are summarized in Table 1.

**TABLE 1:** Baseline characteristics of included literatures.

Study	Type of patients	Sample Size		Age (years, mean±SD)		Gender (male/female)		Drugs			Follow-up (months)
		Intervention	Control	Intervention	Control	ARNI	Control	Intervention	Control	Initial time	
Chen et al. (2019)	AMI PPCI HF	26	26	56.3 ± 10.1	55.7 ± 9.7	15/11	14/12	sacubitril–valsartan 200 mg bid	Enalapril 5 mg qd	In 2 weeks after PPCI	6
Chen et al. (2020)	AMI PPCI HFrEF	30	30	55.4 ± 10.1	54.6 ± 10.3	15/15	15/15	Sacubitril–valsartan MTD	Enalapril MTD	After PPCI	6
Wang et al. (2020)	STEMI PPCI HFrEF	80	80	59.0 ± 10.3	58.0 ± 10.4	69/11	67/13	Sacubitril–valsartan MTD	Valsartan MTD	In 1 week after PPCI	6
Zhao (2020)	STEMI PPCI HFrEF	62	61	68.2 ± 2.4	68.4 ± 2.3	39/23	40/21	Sacubitril–valsartan MTD	Valsartan MTD	In 1 week after PPCI	6
Li et al. (2020)	AMI PPCI	50	50	54.4 ± 5.9	54.9 ± 6.1	28/22	27/23	Sacubitril–valsartan 200 mg bid	Enalapril 10 mg bid	After PPCI	6
Dong et al. (2020)	STEMI PPCI HFrEF	40	40	63.9 ± 8.2	62.0 ± 7.6	23/17	26/14	Sacubitril–valsartan 200 mg bid	Valsartan 80 mg qd	After PPCI	6
Zhao et al. (2020)	AMI PPCI HFrEF	45	45	62.8 ± 3.9	63.2 ± 4.6	25/20	27/18	Sacubitril–valsartan 50 mg bid	Valsartan 80 mg qd	In 24 h after PPCI	3
Wang et al. (2020)	STEMI PPCI HFrEF	68	69	59.1 ± 7.2	60.6 ± 7.6	52/16	54/15	Sacubitril–valsartan 100 mg bid	Enalapril 5 mg bid	After PPCI	6
Zhang et al. (2021a)	STEMI PPCI	79	77	60.3 ± 11.7	60.0 ± 10.9	59/20	55/22	Sacubitril–valsartan MTD	Perindopril MTD	In 24 hours after PPCI	6
Rezq et al. (2021)	STEMI PPCI	100	100	52.0 ± 9.2	57.0 ± 11.6	86/14	88/12	Sacubitril–valsartan 100 mg bid	Ramipril 5 mg bid	After PPCI	6
Zhang et al. (2021a)	AMI PPCI HFrEF	43	43	48.6 ± 10.4	49.8 ± 7.2	35/8	37/6	Sacubitril–valsartan 200 mg bid	Benazepril 10 mg qd	After PPCI	3
Chen (2021)	AMI PPCI HFrEF	31	30	56.3 ± 10.1	55. 7 ± 9.7	16/15	17/13	Sacubitril–valsartan 100 mg qd	Enalapril 5 mg qd	After PPCI	1
Zhang et al. (2021c)	AMI PPCI	56	67	51.9 ± 3.4	52.8 ± 3.3	36/20	39/18	Sacubitril–valsartan 200 mg bid	Enalapril 20 mg qd	After PPCI	6
Yang et al. (2021)	AMI PPCI	38	38	60.0 ± 13.0	55.0 ± 12.0	31/7	35/3	Sacubitril–valsartan 100 mg bid	Valsartan 80 mg qd	After PPCI	3
Gu (2021)	AMI PPCI HF	40	40	62.2 ± 5.4	62.2 ± 4.5	24/16	22/18	Sacubitril–valsartan MTD	Valsartan MTD	After PPCI	6
Fu and Xu (2022)	STEMI PPCI HFrEF	63	63	64.1 ± 9.1	67.4 ± 11.5	44/19	47/16	Sacubitril–valsartan 200 mg bid	Fosinopril 40 mg qd	After PPCI	6
Li and Xiong (2022)	STEMI PPCI HFrEF	50	48	62.4 ± 9.8	62.7 ± 10.1	27/23	26/22	Sacubitril–valsartan 200 mg bid	Perindopril MTD	after PPCI	6
Liu et al. (2022)	AMI PPCI	165	183	58.1 ± 10.2	58.0 ± 11.7	129/36	150/33	Sacubitril–valsartan 200 mg bid	Ramipril 10 mg qd	After PPCI	6
Yang et al. (2022b)	STEMI PPCI HFrEF	48	47	55.8 ± 11.4	56.2 ± 10.9	41/7	38/9	Sacubitril–valsartan MTD	Valsartan MTD	After PPCI	6
Ma and Yang (2022)	AMI PPCI HFrEF	30	30	62.4 ± 5.6	63.3 ± 6.1	18/12	16/14	Sacubitril–valsartan 200mg bid	Enalapril/ valsartan MTD	After PPCI	6
Dong et al. (2022)	STEMI PPCI	65	66	60.2 ± 9.8	60.4 ± 10.0	51	53	Sacubitril–valsartan 200 mg bid	Enalapril 10 mg bid	In 24 h after PPCI	6

Notes: AMI, acute myocardial infraction; HF, heart failure; HFrEF, heart failure with reduced ejection fraction; PPCI, Primary percutaneous coronary intervention; MTD, maximum tolerated dose; SD, standard deviation; STEMI, ST segment elevation myocardial infarction.

### Risk of bias assessment and publication bias

The quality of the included 21 RCTs was shown in Supplementary Table S1 and Supplementary Figure S1. Of the included studies, the overall quality of the included literature is relatively high, but a risk of bias could be found as well. For most of the studies, the funnel plots were symmetric, and the *p*-value for the Egger test was less than 0.05, except for NT-proBNP. The funnel plot for NT-proBNP was not symmetric, and the *p*-value for the Egger test was less than 0.05 (*p* = 0.01), indicating that there was a publication bias in this study.

## Primary outcomes

### Echocardiographic indexes related to left ventricular remodeling

**FIGURE 2:**
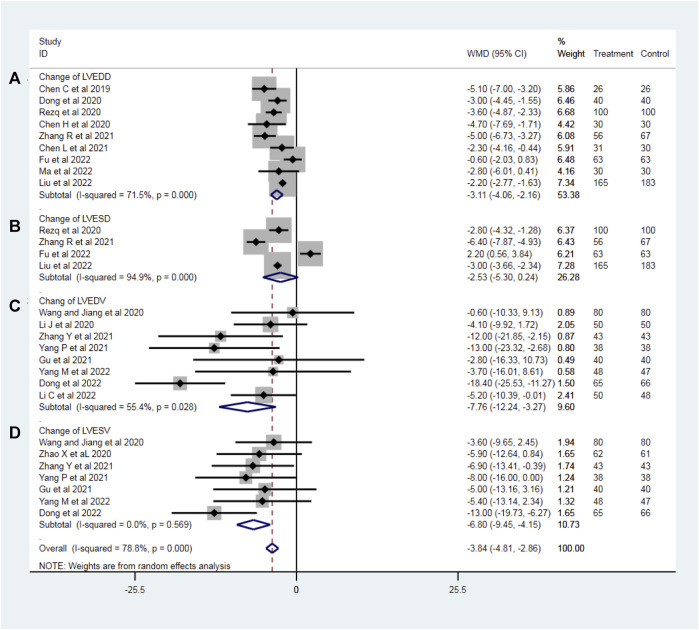
Change of LVEDD **(A)**, LVESD **(B)**, LVEDV **(C),** and LVESV **(D)** with sacubitril–valsartan vs. ACEI/ARB in AMI patients after PPCI. LVEDD, left ventricular end-diastolic dimension; LVESD, change of left ventricular end-systolic dimension; LVEDV, left ventricular end-diastolic volume; LVESV, left ventricular end-systolic volume. ACEI, angiotensin-converting enzyme inhibitor; ARB, angiotensin receptor blocker.

To evaluate the effect of sacubitril–valsartan on left ventricular remodeling in AMI patients after PPCI, we analyzed the change of the most common echocardiographic indexes related to left ventricular remodeling including LVEDD (nine RCTs with 1,110 patients), LVESD (four RCTs with 797 patients), LVEDV (eight RCTs with 826 patients) and LVESV (seven RCTs with 751 patients). No heterogeneity was found in the results of LVESD (*I* ^*2*^ = 0%), the others had moderate to considerable heterogeneity (LVEDD: *I* ^*2*^ = 71.5%; LVESD: *I* ^*2*^ *=* 94.9%; LVEDV: *I* ^*2*^ *=* 55.4%) and a random effect model was used in this part accordingly. Compared with the ACEI/ARB group, the sacubitril–valsartan treatment reversed the LVEDD in AMI patients after PPCI (WMD −3.11, 95%CI: −4.05∼−2.16, *p* < 0.001; Figure 2A). Similarly, sacubitril–valsartan treatment reduced the LVESD in AMI patients after PPCI, but the reduction was not statistically significant (WMD −2.53, 95%CI: −5.30–0.24, *p* = 0.074; Figure 2B). In terms of left ventricular volume change, sacubitril–valsartan successfully reduced LVEDV in those specific patients (WMD −7.76, 95%CI: −12.24∼−3.27, *p* = 0.001; Figure 2C). Meanwhile, similar effects were also observed in the change of LVESV (WMD −6.80, 95%CI: −9.45∼−4.15, *p* < 0.001; Figure 2D).

**FIGURE 3:**
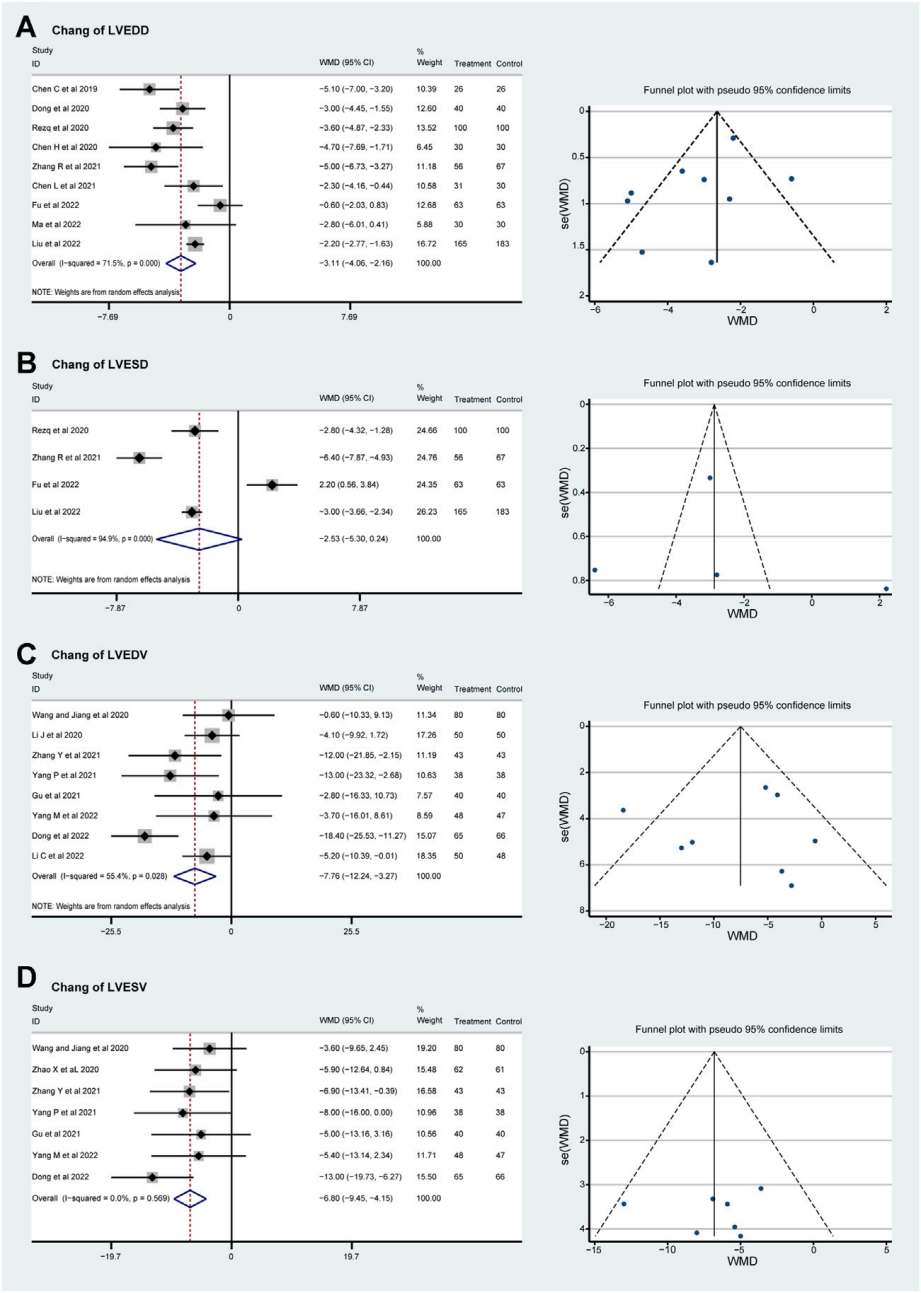
Funnel plots and subgroup analyses based on sacubitril–valsartan dosage, comparing its effects to those of ACEI/ARB in AMI patients after PPCI. **(A)** LVEDD, **(B)** LVESD, **(C)** LVEDV, and **(D)** LVESV. ACEI, angiotensin-converting enzyme inhibitor; ARB, angiotensin receptor blocker.

Subgroup analyses were conducted according to the dosage of sacubitril–valsartan. Though the heterogeneity of LVEDD can be detected as well, it was decreased slightly, and the result stayed stable, showing that LVEDD can be reversed (WMD_smaller dose_ −3.14, 95%CI: −4.36∼−1.92, *p* = 0.258 and WMD_larger dose_ −3.18, 95%CI: −4.40∼−1.95, *p* < 0.001; Figure 3A). The value of left ventricular end-systolic dimension (LVESD) was decreased with high heterogeneity in patients taking higher doses of sacubitril–valsartan (WMD_larger dose_ −2.43, 95%CI: −6.27–1.42, *p* < 0.001); only Rezq et al., 2020 was included in the smaller dosage group (Figure 3B). The direction for LVEDV was similar, supporting our conclusion (WMD_smaller dose_ −7.76, 95%CI: −12.24∼−3.27, *p* = 0.028 and WMD_larger dose_ −7.12, 95%CI: −11.99∼−2.26, *p* = 0.024; Figure 3C). Moreover, the funnel plots proved that the result was convincing. No heterogeneity was found in the LVESV group. Subgroup analysis indicated that the drug could reverse LVESV (WMD_smaller dose_ −8.00, 95%CI: −16.00∼−3.27, *p* < 0.001 and WMD_larger dose_ −6.65, 95%CI: −9.46∼−3.85, *p* = 0.453; Figure 3D).

### Incidence of MACE, myocardial reinfarction, and heart failure

**FIGURE 4:**
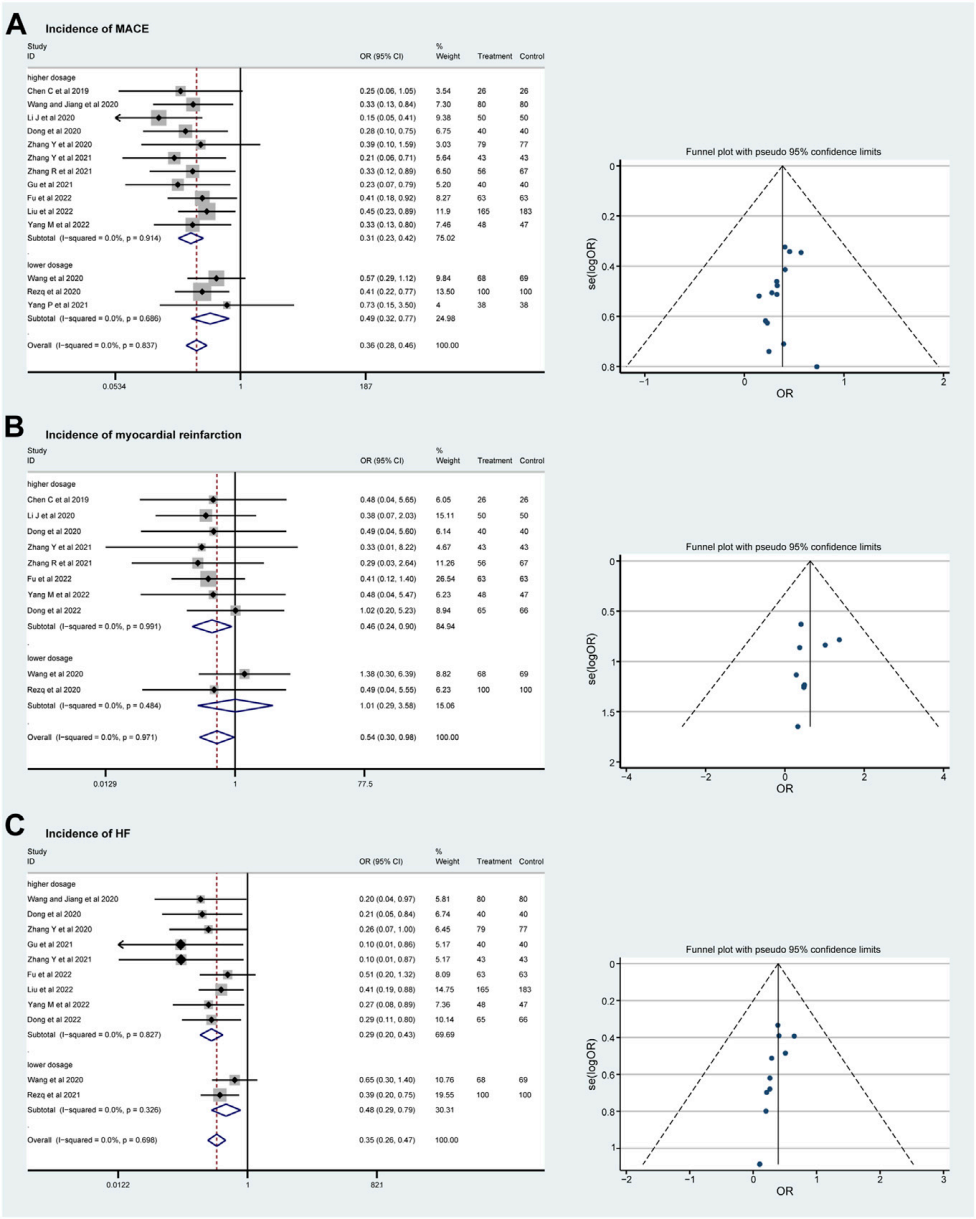
Funnel plots and subgroup analyses comparing the risks of MACE **(A)**, myocardial reinfarction **(B),** and HF **(C)** with sacubitril–valsartan vs. ACEI/ARB in AMI patients after PPCI. ACEI, angiotensin-converting enzyme inhibitor; ARB, angiotensin receptor blocker. MACE, major adverse cardiac event; HF, heart failure.

No significant heterogeneity was found in the incidence of MACE, myocardial reinfarction, and HF (*I* ^*2*^ = 0%), and a fixed effect model was used for meta-analysis. In total, fourteen RCTs with 1819 patients evaluated the incidence of MACE during follow-up. Both the lower and the higher dosage groups show a lower incidence of MACE (OR = 0.49, 95%CI: 0.32–0.77, *p* = 0.686 and OR = 0.31, 95%CI: 0.23–0.42, *p* = 0.914; Figure 4A). The incidence rate of myocardial reinfarction was explored in ten RCTs that included 1,130 patients. The incidence of myocardial reinfarction was lower in the higher-dosage sacubitril–valsartan group (OR = 1.01, 95%CI: 0.29–3.58, *p* = 0.484 and OR = 0.46, 95%CI: 0.24–0.90, *p* = 0.991; Figure 4B). In addition, the incidence rate of HF after treatment was evaluated in eleven RCTs that included 1,605 patients. The two dosage groups share a similar trend of lower incidence of HF after treatment (OR = 0.48, 95%CI: 0.29–0.79, *p* = 0.326 and OR = 0.29, 95%CI: 0.20–0.43, *p* = 0.827; Figure 4C). As for total effects, the incidence rate of MACE, myocardial reinfarction, and HF after PPCI were decreased significantly (OR = 0.36, 95%CI: 0.28–0.46, *p* < 0.001, OR = 0.54, 95%CI: 0.30–0.98, *p* = 0.041 and OR = 0.35, 95%CI: 0.26–0.47, *p* < 0.001 separately).

## Secondary outcomes

### NT-proBNP level

**FIGURE 5:**
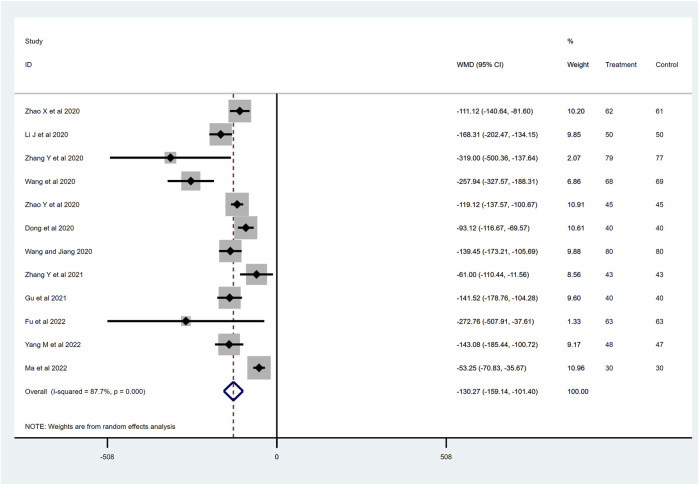
NT-proBNP level with sacubitril–valsartan vs. ACEI/ARB in AMI patients after PPCI. NT-proBNP, N-terminal pro-brain natriuretic peptide. ACEI, angiotensin-converting enzyme inhibitor; ARB, angiotensin receptor blocker.

After removing five studies that may have contributed to significant heterogeneity in the sensitivity analysis, we included twelve RCTs with 1,293 patients that reported the NT-proBNP level at the time of the last visit. The heterogeneity (*I* ^*2*^ = 87.7%) of the included articles was considerable, and a random effect model was used. Compared with the ACEI/ARB group, sacubitril–valsartan treatment significantly decreased the NT-proBNP level in AMI patients after PPCI (WMD −130.27, 95%CI: −159.14∼−101.40, *p* < 0.001; Figure 5). NT-proBNP is a biological marker that could diagnose heart failure, and the decrease in this value indicates that the cardiac load is low or that myocardial function is in a relatively good state.

### Change of LVEF

**FIGURE 6:**
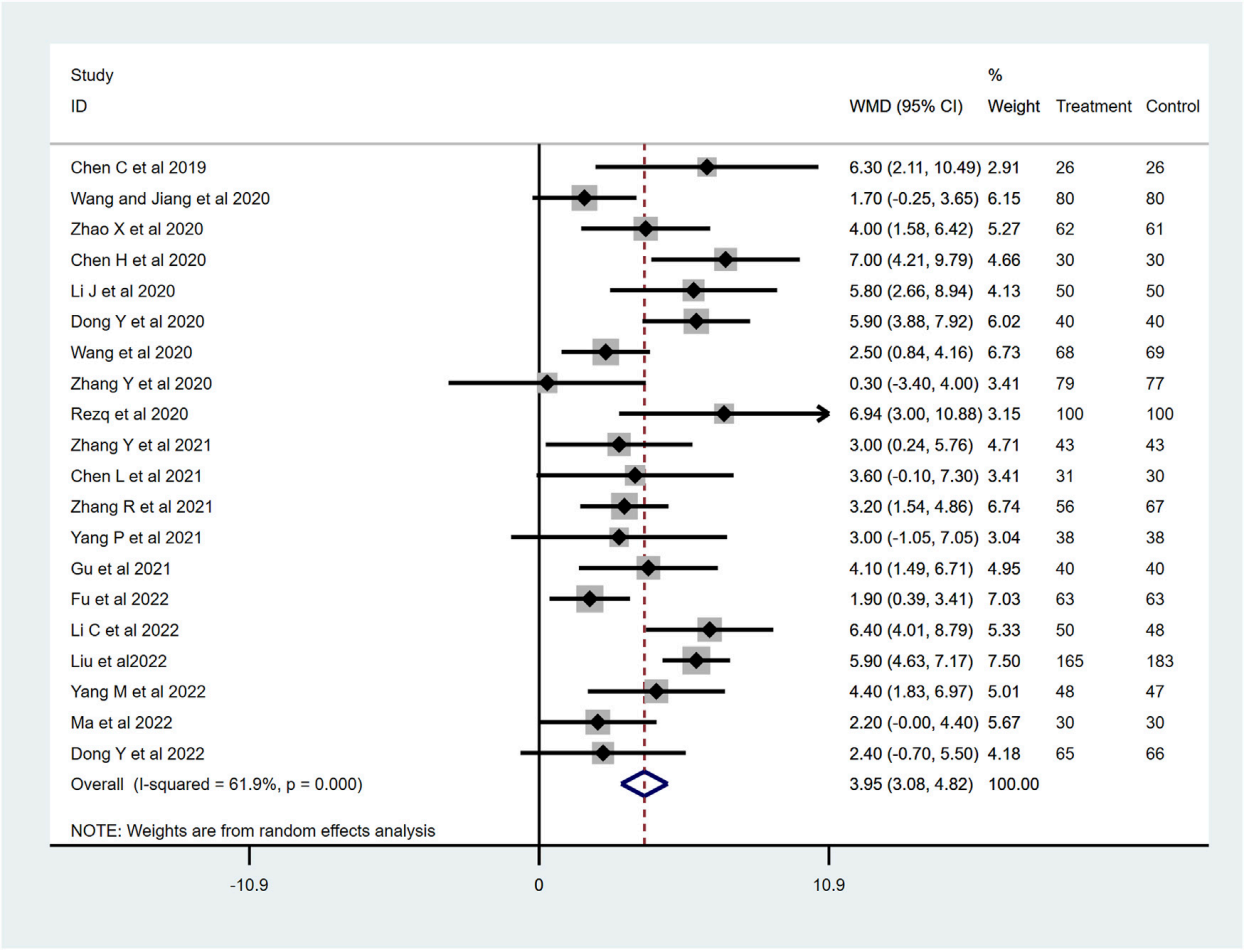
Change of LVEF with sacubitril–valsartan vs. ACEI/ARB in AMI patients after PPCI. LVEF, left ventricular ejection fraction. ACEI, angiotensin-converting enzyme inhibitor; ARB, angiotensin receptor blocker.

LVEF changes were reported in twenty-one RCTs. When we excluded Zhao et al. (2020) in the sensitive analyses, the heterogeneity decreased from 85.8% to 61.9%. We included twenty studies consisting of 2,326 patients to examine the effects of sacubitril–valsartan on LVEF. A random-effect model was applied, and the final result showed that the use of the sacubitril–valsartan improves the LVEF (WMD 3.91, 95%CI: 3.41–4.41, *p* < 0.001; Figure 6). LVEF is closely associated with cardiac function. A stable value typically indicates that there has been no significant impairment or decline in left ventricular function for AMI patients undergoing PPCI.

### 6-min walk test (6MWT) distance

**FIGURE 7:**
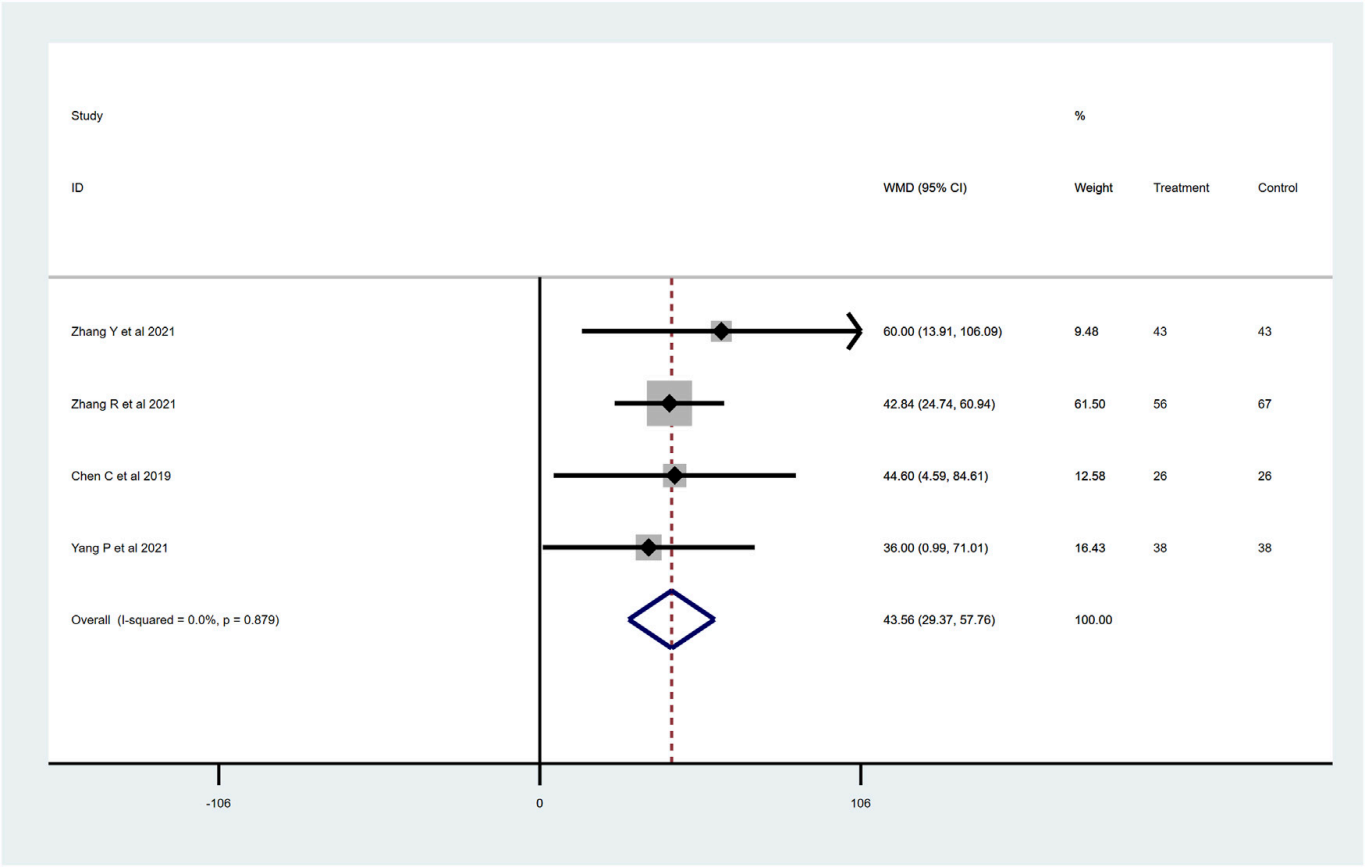
6MWT distance with sacubitril–valsartan vs. ACEI/ARB in AMI patients after PPCI. 6MWT, 6-min walk test. ACEI, angiotensin-converting enzyme inhibitor; ARB, angiotensin receptor blocker.

To determine the effects of sacubitril–valsartan on 6MWT, four RCTs with 337 patients were included after removing Zhao et al. (2020), which greatly influenced the heterogeneity of the study (*I* ^*2*^ = 95.0%). The remaining four RCTs showed no heterogeneity, and the final results show improvement in the 6MWT in the patients who received sacubitril–valsartan (WMD 43.56, 95%CI: 29.37–57.76, *p* < 0.001; Figure 7). Generally, an improvement in the distance walked during the test indicates an improvement in exercise tolerance and cardiovascular function.

### Incidence of adverse drug reactions

**FIGURE 8:**
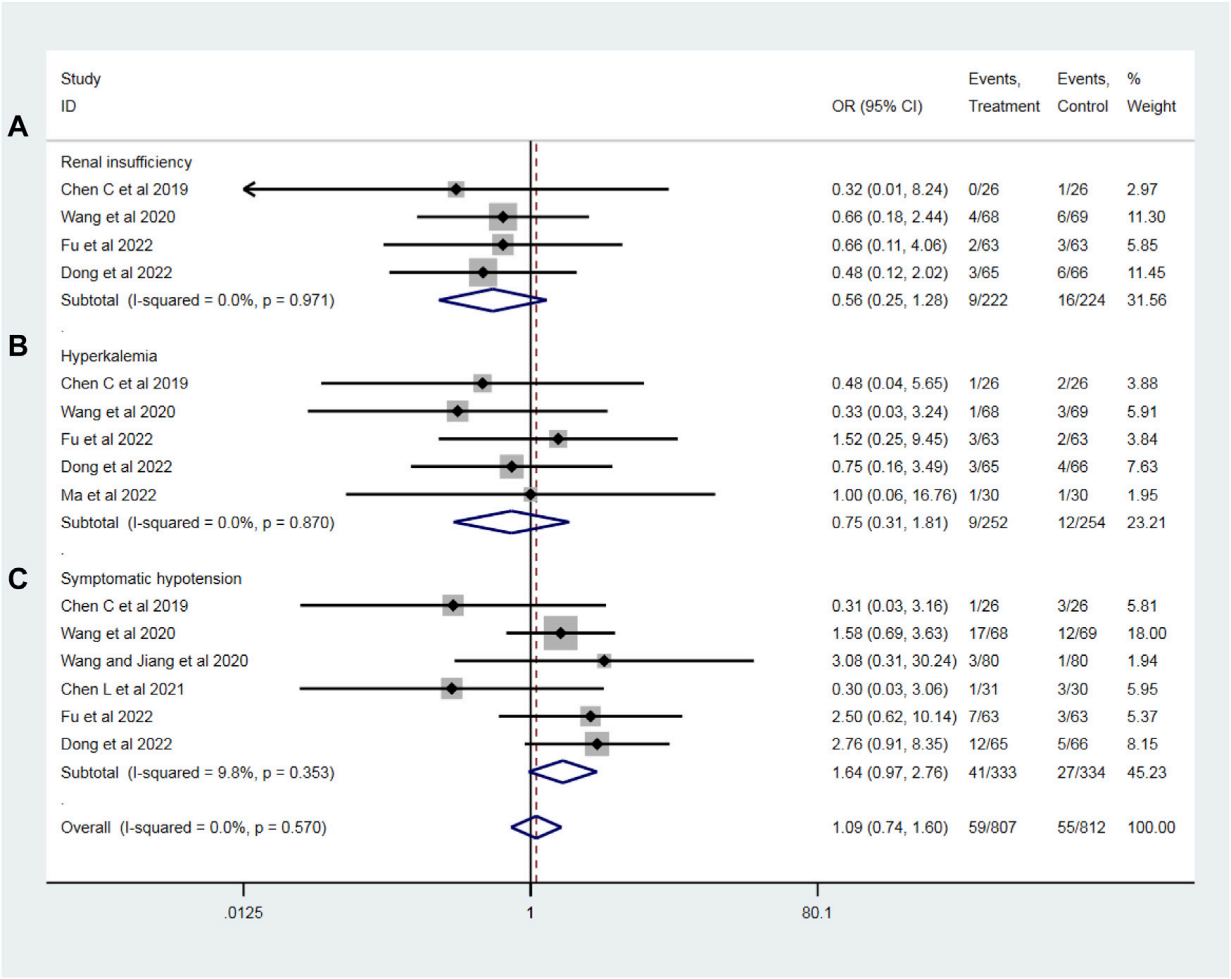
Risks of adverse drug reactions, including renal insufficiency **(A)**, hyperkalemia **(B)**, and symptomatic hypotension **(C)** with sacubitril–valsartan vs. ACEI/ARB in AMI patients after PPCI. ACEI, angiotensin-converting enzyme inhibitor; ARB, angiotensin receptor blocker.

To evaluate the drug safety after PPCI, we analyzed the most common adverse drug reactions of sacubitril–valsartan and ACEI/ARB, including renal insufficiency (four RCTs with 446 patients), hyperkalemia (five RCTs with 506 patients) and symptomatic hypotension (six RCTs with 667 patients) during follow-up. All the *I* ^*2*^ values of the above-mentioned outcomes were less than 25%, indicating no significant heterogeneity, so a fixed effect model was used. Compared with the ACEI/ARB group, sacubitril–valsartan treatment did not significantly increase the incidence of renal insufficiency (OR = 0.56, 95%CI: 0.25–1.28, *p* = 0.170; Figure 8A), hyperkalemia (OR = 0.75, 95%CI: 0.31–1.81, *p* = 0.517; Figure 8B), or symptomatic hypotension (OR = 1.64, 95%CI: 0.97–2.76, *p* = 0.064; Figure 8C) in patients with AMI after PPCI.

## Discussion

For the treatment of AMI, timely PPCI can complete IRA reperfusion and rescue ischemic myocardium. However, increasing evidence suggests that some AMI patients accepting PPCI for revascularization still develop left ventricular remodeling (Andreas et al., 2022). Cardiac remodeling post-AMI is influenced by cardiac stretching, neurohormonal activation, paracrine and/or autocrine factors, and RAAS activation (Jun et al., 2023). Furthermore, pathological left ventricular remodeling post-myocardial infarction is correlated to the risk of HF. Some studies reported that HF is a consequence of cardiomyocyte death and scar formation occurring alongside left ventricular remodeling (Thomas and Rajesh, 2017). Therefore, myocardial injury and ventricular remodeling exert influence on each other (Pfeffer and Braunwald, 1990). Sacubitril–valsartan can improve cardiac structure, systolic function, and LVEF by reversing cardiac remodeling to improve the prognosis of patients with HF (Henan et al., 2024). This is the reason why we chose sacubitril–valsartan as the research drug, hoping to improve left ventricular remodeling after PPCI in AMI patients.

LVEDV and LVEDD are indicators of left ventricular end-diastolic volume and diameter, used to assess the size and diastolic function of the left ventricle. They reflect the volume and size of the left ventricle during the cardiac diastole. Typically, an increase in LVEDV and LVEDD may indicate impaired left ventricular diastolic function or issues such as myocardial hypertrophy. In the included trials, a total of 1,110 patients who recorded LVEDD values were included in nine RCTs examining AMI patients after PPCI, and the results showed that compared with the control group, sacubitril–valsartan robustly reduced the LVEDD. Although the *I* ^*2*^ test was>50%, suggesting moderate heterogeneity, according to the funnel plot of the LVEDD, we found that all included studies were mostly symmetrically distributed in the funnel plot, suggesting that the heterogeneity remained within an acceptable range. We believe that the main source of heterogeneity may be due to the different drugs used in control groups, the wide variation in the enrollment conditions, or the lack of uniformity of information about the included participants. The decrease in LVEDV proved that sacubitril–valsartan could reduce the likelihood of left ventricular remodeling as well.

LVESV and LVESD are important parameters that assess the left ventricular ejection function, reflecting the size and volume of the heart during cardiac diastole. Increased LVESV and LVESD are typically associated with impaired left ventricular function or myocardial damage. In this study, sacubitril–valsartan treatment tended to reduce the LVESD and LVESV in AMI patients after PPCI, and the *p*-value was significant for LVESD. Although the *p*-value for LVESV was over 0.05 (*p* = 0.569), it shared the same trend with LVESD.

Taken together, our meta-analysis demonstrated that compared with conventional ACEI/ARB, administration of sacubitril–valsartan significantly alleviated LV remodeling after PPCI in patients with AMI. Similar to our research results, Amil et al. (2022) demonstrated patients with AMI treated with sacubitril–valsartan had less increase in LV end-diastolic volume and more decrease in LV mass index. The drugs’ mechanism of action is that sacubitril–valsartan not only inhibits RAAS but also augments natriuretic peptides by inhibiting their breakdown by neprilysin. Under this dual mechanism of action, sacubitril–valsartan avoids excessive degradation of brain natriuretic peptide and reduces the release of renin, angiotensin, and aldosterone, thus playing a role in vasodilation, promoting the excretion of urine sodium, leading a decrease of cardiac volume and pressure load, ultimately reversing ventricular remodeling (Wilfried and Pieter, 2018; Nicholas et al., 2019). However, our finding was inconsistent with the Kieran et al. (2021) study. Docherty et al. reported that compared with valsartan, sacubitril–valsartan did not significantly reduce the LVEDV index in AMI patients. The main reason for the discrepancy may be related to different initial drug administration times. Generally speaking, various mechanisms, including inflammation, cell apoptosis, and ischemia-reperfusion injury, are involved in the occurrence of ventricular remodeling at the early stage of AMI (Alessandro et al., 2020). Therefore, early drug treatment may be important for reversing ventricular remodeling after AMI. In the Docherty et al. study, all patients started using valsartan or sacubitril–valsartan treatment 3 months after myocardial infarction. During the interval before the drugs were administered, structural remodeling may have occurred, even leading to HF and irreversible myocardial damage. Furthermore, it is difficult to reverse the myocardial damage that has already occurred. In contrast, in the RCTs included in the current meta-analysis, most AMI patients received either sacubitril–valsartan or ACEI/ARB treatment after receiving PPCI for revascularization. Therefore, the difference between our meta-analysis and the study of Docherty et al. may be mainly attributed to the difference in the initial administration time of sacubitril–valsartan. Moreover, the subgroup analyses showed that for most of the studies, the inhibitory effect of sacubitril–valsartan on those left ventricular-related parameters was even more pronounced in the lower dose group. It is possible that perhaps the timely application of a lower dose of ARNI after PPCI may offer optimal effects on ventricular remodeling because the low dose was well tolerated (Jun et al., 2023). However, potential confounding factors such as baseline differences cannot be excluded. For instance, groups receiving higher doses of ARNI may have more comorbidities, such as hypertension. Additionally, only a minority of the studies included in our analysis utilized lower doses of ARNI, which would inevitably introduce bias. However, the positive effects demonstrated by the use of lower doses of ARNI in our study are promising and may potentially become routine treatment for post-PPCI patients with AMI in the future.

It is still controversial whether early administration of sacubitril–valsartan can bring more benefits to patients with AMI after receiving PPCI to complete revascularization. Some studies have suggested the possibility that sacubitril–valsartan can reduce myocardial infarction scar size and the risk of ventricular arrhythmias in myocardial infarction model animals (Jian et al., 2021; Laura, 2021). In the current study, we found that after PPCI, patients with AMI receiving sacubitril–valsartan treatment had a relatively lower incidence of MACE, myocardial reinfarction, and post-PPCI HF compared with the ACEI/ARB group. There is no heterogeneity in this part of the current meta-analysis. MACE comprise cardiovascular (CV) death, nonfatal myocardial infarction, and nonfatal stroke. Reducing the incidence of MACE improves survival rates and quality of life (Naveed et al., 2021). The occurrence of myocardial reinfarction indicates unstable cardiovascular status, potentially linked to inadequate primary infarction treatment, progression of coronary artery disease, or thrombus formation. Myocardial reinfarction exacerbates myocardial damage, increases cardiac load, and further affects cardiac function (Singh and Venkatesh, 1984). The occurrence of HF after AMI typically signifies severe myocardial injury and declining cardiac function, leading to deterioration in cardiac pumping function. Intervention in such cases can enhance patients’ quality of life and improve long-term prognosis (Ijsbrand et al., 2011).

Nevertheless, the PARADISE-MI study suggests completely different research findings from the current study. The PARADISE-MI study is an international, multicenter, double-blind RCT designed to determine whether sacubitril–valsartan is superior to ramipril in reducing cardiovascular risk in patients with AMI. Unfortunately, the PARADISE-MI study showed that compared to ramipril, sacubitril–valsartan did not significantly reduce the incidence of cardiovascular causes of death or HF among patients with AMI. However, the sacubitril–valsartan treatment was shown to be more effective than ramipril in preventing the recurrence of HF after the first one. The different included research subject populations could partly explain this difference. In the PARADISE-MI study, the main inclusion criterion was AMI associated with LVEF≤40% and/or signs of pulmonary congestion evidenced by the use of intravenous diuretics. Moreover, all potential patients were required to meet at least one of the eight additional high-risk criteria (including age≥70 years, diabetes, history of previous myocardial infarction, atrial fibrillation, LVEF <30%, Killip Class ≥ III, STEMI without reperfusion within 24 h, and glomerular filtration rate <60 mL/min/1.73 m^2^). As a result, all enrolled AMI patients in the PARADISE-MI study were high-risk myocardial infarction patients. High-risk myocardial infarction patients have higher Global Registry of Acute *Coronary* Events (GRACE) scores and a higher risk of cardiovascular adverse events, including HF, myocardial reinfarction, and arrhythmia. In addition, some AMI patients in the PARADISE-MI had not even completed IRA early ischemia-reperfusion therapy. In detail, the proportions of patients without coronary reperfusion in the sacubitril–valsartan group and the ramipril group were 11% and 12%, respectively. In contrast, all AMI patients in the included RCTs in the current meta-analysis received PPCI therapy for revascularization. As is well known, if effective coronary artery reperfusion is not performed, resulting in persistent myocardial ischemia, myocardial cell necrosis, and ventricular remodeling, the risk of adverse cardiovascular events post-AMI will increase, especially the risk of HF. Hence, high-risk patients and patients who have not completed coronary reperfusion may have an increased risk of clinical adverse events during the follow-up period and affect research results.

On the other hand, large-scale cardiovascular clinical trials commonly used to improve statistical efficiency often equate the impact of any event occurring in the composite endpoint on prognosis. However, this strategy did not take into account the clinical relevance and severity of the event; instead, it conducted indiscriminate merge analysis. The win ratio analysis was first reported in 2012. When analyzing survival data with multiple outcomes, the importance of outcomes can be considered to address potential biases caused by varying severity of composite endpoints. Moreover, the win ratio method can be stratified based on the importance of outcomes, thereby more objectively evaluating the effectiveness of interventions. Using the win ratio analysis method, the *post hoc* analysis of the PARADISE-MI study showed a larger number of wins than losses in the sacubitril–valsartan group (win ratio of 1.17, 95%CI: 1.03–1.33; *p* = 0.015), suggesting sacubitril–valsartan was superior to ramipril among high-risk survivors of AMI (Otavio et al., 2022). The win ratio analysis of the PARADISE-MI trial provides an additional perspective for understanding the role of sacubitril–valsartan in patients with AMI.

The improvement in the prognosis of sacubitril–valsartan was also reflected in the improvement of LVEF and 6MWT distance after treatment. As is well known, LVEF is a common indicator of cardiac function, and its value is positively correlated with cardiac function. At the same time, the 6MWT distance in the sacubitril–valsartan group after treatment was significantly longer than that of the control group, suggesting that sacubitril–valsartan could significantly improve the activity tolerance of AMI patients after PPCI and improve the cardiac function of patients with chronic HF. The NT-proBNP level decreased significantly after treatment with sacubitril–valsartan, which is consistent with the conclusions of and the PIONEER-HF study, but with high heterogeneity (Berg et al., 2021). Patients with acute HF might have higher baseline levels of NT-proBNP, and they are likely to experience a greater magnitude of reduction in NT-proBNP levels. We could not group our studies by that value due to a lack of original data. Moreover, publication bias might be a possible factor influencing the results. We finally applied a random effects model that assumes that the true effect sizes of different studies are random variables and takes into account the variability between studies, which can, to some extent, reduce the influence of heterogeneity. NT-proBNP is widely used in HF screening and diagnosis (Michael et al., 2016). Natriuretic peptide concentrations can reflect pro-fibrotic environments and could be used to stratify individuals at risk for remodeling, a patient group that currently cannot be adequately assessed by conventional imaging methods (Jens et al., 2020). Interestingly, some evidence suggests injecting recombinant human BNP prior to coronary stent implantation appears to confer some degree of protection from myocardial injury, highlighting the therapeutic potential of the recombinant human BNP (Shiqiang et al., 2015). Meanwhile, compared to ACEI/ARB treatment, sacubitril–valsartan after PPCI in patients with AMI did not increase the risk of renal dysfunction, hyperkalemia, or orthostatic hypotension.

## Limitation

The current meta-analysis has several limitations. First, the sample size of some included RCTs in the study was generally small, resulting in bias in the experimental results. Second, HFrEF, HF patients with mid-range ejection fraction (HFmrEF), and HF patients with preserved ejection fraction (HFpEF) were not discussed separately in most of the included RCTs. To our knowledge, the efficacy of sacubitril–valsartan in these populations varied and was inconclusive. Third, the length of the follow-up periods in the included RCTs was unequal. Changes in cardiac structure and function after AMI required a certain amount of time to form, and the time required for some patients to titrate to the highest concentration of drug tolerance in the experiment varied. We tried to group the studies by the length of follow-up, but the numbers of studies in the two groups were not equal, which makes the subgroup analyses less trustworthy.

## Prospective

First, large scales of randomized controlled trials should be designed in the future, with more detailed recording of basic information of the enrolled population, including initiation time of medication, participant comorbidities, and other baseline information, to better ensure the accuracy of experimental results. Second, standardized medication and long-term follow-up are necessary, especially for the evaluation of efficacy and safety. Third, this study emphasizes that timely use of relevant drugs in patients with AMI undergoing PPCI can improve ventricular remodeling, decrease the rate of heart failure, and improve long-term prognosis. Future studies will focus on investigating whether ARNI can be used as a foundational medication to guide treatment after PPCI.

## Conclusion

In conclusion, our meta-analysis indicates that compared with ACEI/ARB, sacubitril–valsartan may be superior to reverse left ventricular remodeling, improve cardiac function, and effectively reduce the risk of MACE, myocardial reinfarction, and HF in AMI patients during follow-up without increasing the risk of adverse reactions including renal insufficiency, hyperkalemia, and symptomatic hypotension after PPCI. So, early administration of sacubitril–valsartan after PPCI for AMI patients may be an important treatment option. Our research explored potential drugs for those specific populations to improve left ventricular remodeling, which broadens the application of sacubitril–valsartan and sheds light on promising directions for future research. However, due to the quality and quantity of the included articles, as well as the risk of bias, its efficacy could be overestimated. It needs to be further confirmed by high-quality prospective randomized controlled research to provide corroborating evidence.

## Data Availability

The original contributions presented in the study are included in the article/Supplementary Material; further inquiries can be directed to the corresponding author.
