# Supplementary material for: Effect of sacubitril–valsartan on left ventricular remodeling in patients with acute myocardial infarction after primary percutaneous coronary intervention: a systematic review and meta-analysis
Source: Front Pharmacol. 2024 May 28;15:1366035. doi: 10.3389/fphar.2024.1366035 (PMC11165101; doi:10.3389/fphar.2024.1366035)
Supplement: Supplementary file 1 [file Table1.docx]

**Table S1. Detailed Risk Bias Assessment Of All The Included Trials Using The Cochran Risk Of Bias Tools**

|  | **Selection Bias** | | **Performance Bias** | **Detection Bias** | **Attrition Bias** | **Reporting Bias** |
| --- | --- | --- | --- | --- | --- | --- |
| **Bias Type** | **Random sequence generation** | **Allocation concealment** | **Blinding of participants and personnel** | **Blinding of outcome  assessment** | **Incomplete outcome  data** | **Selective reporting** |
| Chen C-2019^22^ | Low risk | Low risk | Unclear risk | Unclear risk | Low risk | Low risk |
| Chen H-2020^23^ | Low risk | Low risk | Low risk | Unclear risk | Low risk | High risk |
| Wang and Jiang-2020^24^ | Unclear risk | Unclear risk | Unclear risk | Unclear risk | Low risk | Low risk |
| Zhao X-2020^25^ | Low risk | Low risk | Unclear risk | Unclear risk | Low risk | Low risk |
| Li J-2020^26^ | Low risk | Low risk | Unclear risk | Unclear risk | Low risk | Low risk |
| Dong Y-2020^27^ | Unclear risk | Unclear risk | Unclear risk | Unclear risk | Low risk | Low risk |
| Zhao Y-2020^28^ | Low risk | Low risk | High risk | Unclear risk | Unclear risk | High risk |
| Wang H-2020^29^ | Unclear risk | Unclear risk | Unclear risk | Unclear risk | Low risk | Low risk |
| Zhang Y-2020^30^ | Low risk | Low risk | Low risk | Low risk | Low risk | Low risk |
| Rezq-2020^31^ | Low risk | Low risk | Low risk | Low risk | Low risk | Low risk |
| Zhang Y-2021^32^ | High risk | High risk | Unclear risk | Unclear risk | Low risk | Low risk |
| Chen L-2021^33^ | Low risk | Low risk | Unclear risk | Unclear risk | Unclear risk | High risk |
| Zhang R-2021^34^ | High risk | High risk | Unclear risk | Unclear risk | Low risk | Low risk |
| Yang P-2021^35^ | Low risk | Low risk | Unclear risk | Unclear risk | Low risk | Low risk |
| Gu-2021^36^ | Low risk | Low risk | Unclear risk | Unclear risk | Low risk | Low risk |
| Fu-2022^37^ | Low risk | Low risk | High risk | High risk | Low risk | Low risk |
| Li C-2022^38^ | Low risk | Unclear risk | High risk | High risk | Low risk | High risk |
| Liu-2022^39^ | High risk | High risk | Unclear risk | Unclear risk | Unclear risk | Unclear risk |
| Yang M-2022^40^ | Low risk | Low risk | Unclear risk | Unclear risk | Low risk | Low risk |
| Ma-2022^41^ | Low risk | Low risk | Low risk | Low risk | Low risk | Low risk |
| Dong Y-2022^42^ | Low risk | Low risk | Low risk | Low risk | Low risk | Low risk |
