# Supplementary figures and images for: Effect of sacubitril–valsartan on left ventricular remodeling in patients with acute myocardial infarction after primary percutaneous coronary intervention: a systematic review and meta-analysis
Source: Front Pharmacol. 2024 May 28;15:1366035. doi: 10.3389/fphar.2024.1366035 (PMC11165101; doi:10.3389/fphar.2024.1366035)

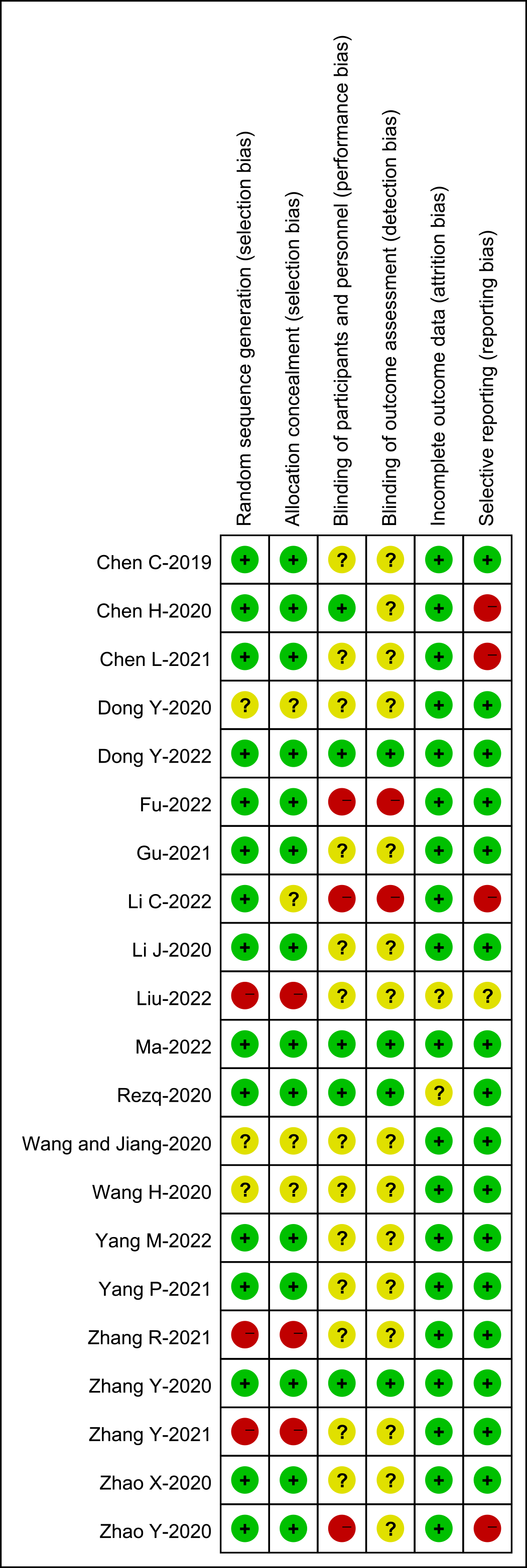

Supplement: Supplementary file 2 [file Image1.TIF]
